# Effects of Sol–Gel Sealing on Corrosion Behavior for MAO White Thermal Control Coating on MB15 Magnesium Alloy

**DOI:** 10.3390/ma19122671

**Published:** 2026-06-22

**Authors:** Jingying Bai, Chen Wen, Jingkang Zhong, Kuo Zhao, Dongcheng Yang, Zishuo Zhang, Xianhua Chen

**Affiliations:** 1 School of Materials Science, Chongqing University, Chongqing 400044, China; baijy1213@163.com; 2 Beijing Xingchi Hengdong Technology Development Co., Ltd., Beijing 100090, China; 15955253681@163.com (J.Z.); fyyzhao@126.com (K.Z.); ydc0218@163.com (D.Y.); zhangzishuo0820@163.com (Z.Z.); 3 Beijing Spacecrafts Manufacturing Co., Ltd., Beijing 100090, China

**Keywords:** corrosion, sol–gel, thermal control coating, MB15, magnesium alloy

## Abstract

**Highlights:**

- A novel sol–gel/micro-arc oxidation (MAO) bilayer retains the coating’s intrinsic thermal control properties while significantly extending service life in harsh, high-salt, high-humidity environments.
- The dual-layer synergistic effect effectively seals inherent MAO micro-defects (pores and microcracks), forming a robust physical barrier against corrosive media.
- Electrochemical impedance spectroscopy (EIS) and scanning electron microscopy (SEM) confirm that the composite coating effectively prolongs the corrosion product formation period relative to single-layer MAO.
- Preserving initial thermal control performance while delivering superior corrosion protection, this bilayer strategy represents a promising solution for aerospace thermal control materials.

**Abstract:**

With the aim of achieving outstanding thermal control and corrosion resistance properties, a white MAO thermal control coating sealed by a silicon–zirconium hybrid sol–gel layer was prepared in this work. The corrosion behavior of the coating was evaluated using potentiodynamic polarization and electrochemical impedance spectroscopy (EIS) in 3.5 wt.% NaCl solution. Microstructural and compositional characterizations were conducted using scanning electron microscopy (SEM), X-ray diffraction (XRD), and energy-dispersive spectroscopy (EDS). Results indicated that the sol–gel/MAO composite coating significantly outperformed the single-layer MAO coating in corrosion resistance, primarily due to effective sealing of micro-pores and cracks by the sol–gel layer, which prevented the penetration of corrosive agents. The post-immersion morphological observations were in good agreement with the EIS results. After immersion, the corrosion current density of the composite coating only increased from 10^−6.4^ to 10^−5.1^ A/cm^2^, while the corrosion potential decreased from −1.25 V to −1.35 V. The post-immersion morphological observations were consistent with EIS results. Meanwhile, the composite coating can effectively mitigate the thermal control performance degradation caused by corrosion. Compared with the MAO coating, the absolute increase in solar absorptance of the sol–gel/MAO coating is reduced by 60%. After 168 h of accelerated corrosion tests in a simulated marine environment, the solar absorptance (α_S_) of the sol–gel/MAO coating increased by only 0.05. This study demonstrates that the combination of MAO and sol–gel treatment provides a promising strategy for the development of lightweight, corrosion-resistant magnesium alloys for aerospace applications.

## 1. Introduction

Magnesium alloys have attracted significant attention due to their low density, high specific strength, and excellent biocompatibility, demonstrating extensive application prospects in the aerospace, automotive, and biomedical fields [1,2,3,4]. However, the high chemical reactivity of magnesium renders it prone to electrochemical corrosion in aggressive environments, which severely limits its broader engineering applications [5]. To endow magnesium alloys with relatively favorable thermal regulation performance and improve their corrosion resistance, micro-arc oxidation (MAO) has been widely employed. This process improves corrosion and wear resistance by generating a dense ceramic oxide coating on the alloy surface [6,7]. Nevertheless, conventional MAO coatings have inherent limitations, such as micro-pores and micro-cracks formed during the rapid melting and solidification processes. These structural defects can serve as pathways for the infiltration of corrosive media, thereby compromising the long-term protective efficiency of the coating [8,9]. This issue is particularly critical in aerospace applications. In such applications, magnesium alloy components are often exposed to extreme conditions like high humidity and salt spray, which accelerate coating degradation [10]. Consequently, optimizing the microstructure of MAO coatings to improve their protective performance has become a key focus of current research.

In pursuit of this goal, the sol–gel method has been proposed as a promising post-treatment technique for modifying micro-arc oxidation (MAO) coatings. This process involves the conversion of inorganic or organic precursors into uniform sols, followed by gelation and heat treatment, leading to the formation of a dense nanoscale film on the MAO-coated surface [11,12]. This sol–gel layer not only effectively seals the micro-pores and micro-cracks within the MAO coating but also further improves corrosion resistance by incorporating functional nanoparticles, such as SiO_2_ and Al_2_O_3_. Numerous studies have demonstrated that sol–gel treatment significantly reduces the surface roughness of MAO coatings, improves their density and chemical stability, and thereby markedly extends the service life of the coated components in corrosive environments [13,14,15]. Furthermore, the sol–gel method possesses advantages including process simplicity, low cost, and the capability to deposit uniform coatings over large areas, which underscores its great potential for industrial applications.

In recent studies focusing on improving the performance of micro-arc oxidation (MAO) coatings via the sol–gel method, significant progress has been achieved by numerous researchers. For instance, Jie Zhao et al. demonstrated a significant improvement in the corrosion resistance of MAO coatings by incorporating nano-SiO_2_ particles into the sol–gel layer and reported electrochemical impedance values more than twice those of the unmodified coating [16]. Xiaowen Chen et al. investigated the effects of different sol–gel formulations on MAO coatings and found that coatings derived from an Al_2_O_3_ precursor exhibited excellent antioxidant properties under high-temperature conditions [17]. Furthermore, Linan Jia et al. and X. W. Chen et al. successfully fabricated multilayer composite films on MAO-coated substrates via the sol–gel technique, which significantly improved both the mechanical properties and corrosion resistance of the coatings [18,19]. These studies not only confirm the effectiveness of the sol–gel method in modifying MAO coatings but also provide a valuable theoretical and experimental basis for further research. In summary, as an efficient and versatile post-treatment technology, the sol–gel method provides innovative strategies and feasible solutions for improving the overall performance of magnesium alloy MAO coatings, indicating broad potential for future applications.

Despite these advancements, research on the application of sol–gel sealing for specific functional MAO coatings, such as white thermal control coatings, remains limited. Notably, most previous studies have primarily focused on enhancing the corrosion resistance of MAO coatings, while they have neglected the critical issue of thermal control performance degradation induced by corrosion. Thermal control performance is a key performance indicator for aerospace components operating in coastal environments. The novelty of this work lies in its dual-objective design, which simultaneously addresses the corrosion vulnerability of white MAO thermal control coatings and protects their inherent thermal control properties from deterioration under high-salt and high-humidity conditions. Therefore, this study aims to develop a low-temperature sol–gel sealing process as an effective post-treatment for micro-arc oxidation (MAO) coatings, which is specifically applied to white thermal control coatings to improve their corrosion resistance. The corrosion behavior of the treated coatings was systematically investigated in a 3.5 wt% NaCl solution. This solution was employed to simulate the service environment of coastal launching sites, with the aim of improving the corrosion resistance of the coatings under high-salinity and high-humidity conditions. The degradation mechanisms were evaluated using scanning electron microscopy (SEM), energy-dispersive X-ray spectroscopy (EDS), X-ray diffraction (XRD), ultraviolet–visible spectroscopy (UV-Vis), and electrochemical impedance spectroscopy (EIS). Based on the electrochemical characteristics, the corrosion mechanisms are comprehensively discussed.

## 2. Experimental

### 2.1. Materials and Specimens

Commercial MB15 magnesium alloy was purchased from Kangji Magnesium Industry Co., Ltd., Dongguan, China. The alloy had factory certification, and its chemical composition was verified by inductively coupled plasma optical emission spectrometry (ICP-OES) to ensure batch consistency and traceability. The specific composition of the elements is listed in Table 1. All samples were cut into 40 mm × 40 mm × 2 mm specimens for subsequent treatment. Before coating, substrates were sequentially ground with 200#, 600#, 800#, and 1200# silicon carbide sandpaper to eliminate surface scratches. After polishing, specimens underwent ultrasonic cleaning with deionized water, organic degreasing with anhydrous ethanol and acetone, and air-drying in a dust-free workstation. This pretreatment removed surface contaminants and oxide layers, providing a clean interface for uniform MAO coating growth.

A silicate-based electrolyte was selected for MAO treatment based on literature and process optimization, as it grants MAO coatings better compactness, thermal stability, and adhesion than phosphate or aluminate systems. The electrolyte contained 10 g/L Na_2_SiO_3_, 8 g/L KOH, and 8 g/L NaF. MAO was conducted with a 750 V, 30 A bipolar pulsed power supply, and an intelligent cooling system kept the electrolyte temperature below 35 °C [20,21]. After MAO, specimens were rinsed with deionized water, dried, and labeled as pure MAO coatings for comparison. To fabricate a composite coating with thermal control and corrosion resistance, a sol–gel layer was sprayed on the MAO underlayer. The dual-sol system was designed based on interface bonding and anti-corrosion theories: GPTMS (propyl trimethyl silicane) formed a flexible cross-linked network to improve MAO compatibility, while zirconium n-propoxide built a dense Zr-O-Si skeleton to block corrosive media [22]. All analytical-grade reagents were purchased from Shanghai Macklin Biochemical Co., Ltd. (Shanghai, China) without further purification.

Dual-sol preparation was performed at 25 °C. The first silicon-based sol was prepared by mixing GPTMS, absolute ethanol, and deionized water (1:3:1, 300 r/min, 1 h) and stirring at 300 r/min for 1 h. The second zirconium-based sol was made by mixing absolute ethanol, glacial acetic acid, and zirconium n-propoxide (CAS: 23519-77-9) (3:2:1, 300 r/min, 1 h), with glacial acetic acid controlling zirconium precursor hydrolysis. After aging, the two sols were blended equally and stirred for 0.5 h to obtain a homogeneous composite sol. MAO specimens were horizontally fixed in a closed spraying workstation. A pneumatic spray gun was used with 0.3 MPa air pressure and 40 mm nozzle–specimen distance. Spraying was divided into three cycles to avoid sagging and bubbles. Between cycles, specimens stood for 15 min at 25 °C and 50 ± 5% relative humidity to volatilize solvents and stabilize the gel network, enhancing coating compactness.

After spraying, specimens were cured in a program-controlled oven at 80 °C for 2 h. This low-temperature curing promoted complete sol–gel cross-linking to form a dense network while avoiding MAO layer cracks or delamination. Sol–gel thickness (15–20 μm) was controlled by on-line monitoring with a high-precision thickness gauge and post-verification via FESEM cross-section observation, ensuring specimen consistency.

The final sol–gel/MAO composite specimens (MB15 substrate + MAO layer + sol–gel layer) were labeled uniformly. To ensure data reliability, three parallel specimens were prepared per group, and electrochemical tests were repeated ≥3 times, with average values and standard deviations reported. The 45 °C, 3.5 wt.% NaCl immersion simulated harsh service conditions (industry standard) and room-temperature normalization before electrochemical tests ensured result comparability.

### 2.2. Test Procedure

The white MAO specimens with and without sealing were immersed in accelerated simulated marine environment of 3.5% NaCl solution at 45 °C for different times. And then the specimens with different immersion times were investigated by electrochemical methods in 3.5%NaCl solution at room temperature. The electrochemical measurements were performed by using a conventional three-electrode electrochemical cell on a Princeton 2273 electrochemical system with a specimen of 1 cm in diameter as the working electrode. The platinum electrode was the counter electrode. A saturated calomel electrode (SCE) was used, and all potentials were referred to this electrode. All electrochemical experiments were performed by three specimens. The open circuit potential (OCP) tests were carried out in potentiostat mode for 1800 s. And then the electrochemical impedance spectroscopy (EIS) was performed with a 10 mV sinusoidal perturbation around the free corrosion potential from 100 kHz to 10 mHz; the data were simulated with Zsimpwin 3.60 software. The potentiodynamic polarization (PP) tests were scanned from −0.5 V to 0.5 V at the rate of 5 mV/s.

### 2.3. Characterization

After the immersion tests with different durations, all specimens were fully dried with clean compressed air for subsequent characterization. The surface micromorphology and cross-sectional microstructure of the composite coatings were observed via field emission scanning electron microscopy (FESEM, SU8010, Hitachi, Tokyo, Japan) at an accelerating voltage of 5–10 kV. The equipped energy dispersive X-ray spectroscopy (EDS) was used for qualitative and quantitative analysis of the surface elemental composition, elemental distribution, and relative elemental content of the coatings, with a working distance of 8 mm and a detection time of 60 s. The crystal phase structure and phase composition of the specimens were characterized by X-ray diffraction (XRD, D8 Advance, Bruker, Karlsruhe, Germany) using Cu Kα radiation (λ = 1.5406 Å). The XRD tests were conducted at an operating voltage of 40 kV and an operating current of 40 mA, with a scanning range of 10–90° and a scanning rate of 2°/min. In addition, the optical absorption properties of the samples were measured by a UV-Vis spectrophotometer (UV-2600, Shimadzu, Kyoto, Japan) within the wavelength range of 200–800 nm at a scanning rate of 100 nm/min, and pure air was adopted as the reference baseline throughout the tests. The thickness of the composite coatings was measured using a portable coating thickness gauge (MiniTest 720, EPK, Saarbrücken, Germany). To ensure test accuracy and repeatability, multiple measuring points were uniformly selected on the surface of each specimen for testing, and the average value was taken as the final coating thickness.

## 3. Results and Discussion

### 3.1. Morphologies and Composition of Sol–Gel/MAO Composite Coatings

**Figure 1:**
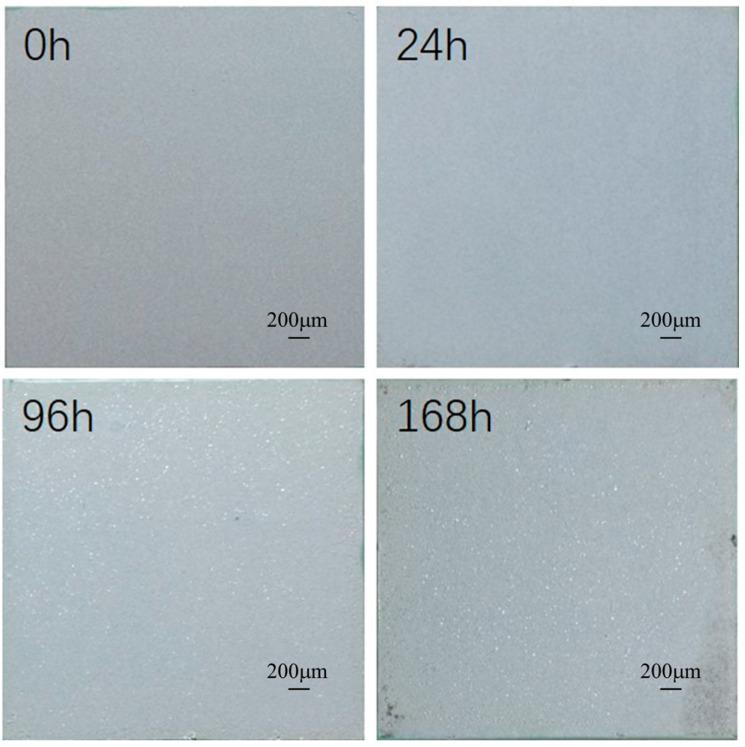
Macroscopic photos of sol–gel/MAO coatings after different immersion times.

In the research on the sealing and corrosion resistance of magnesium alloy micro-arc oxidation (MAO) coatings, the sol–gel technology, as an effective post-treatment method, can significantly improve the corrosion resistance of the coating. This study experimentally investigated the effect of sol–gel sealing treatment on the corrosion behavior of magnesium alloy MAO coatings in a 3.5% sodium chloride solution. Firstly, the morphological changes in the sol–gel composite film layer under different soaking times (0 h, 24 h, 96 h, 168 h) were focused on, and the corresponding macroscopic morphological changes are shown in Figure 1 [23,24].

The experimental results showed that after 24 h of soaking, the macroscopic state of the coating did not undergo significant changes, indicating that the sol–gel sealing layer could effectively prevent the penetration of corrosive media in the initial stage and maintain the integrity of the coating. However, after 96 h of soaking, obvious corrosion phenomena appeared on the coating surface, manifested as local peeling and color changes. This might be due to the gradual failure of the sol–gel sealing layer after long-term soaking, allowing the corrosive media to penetrate into the MAO coating interior and causing corrosion of the magnesium alloy substrate [19]. It is worth noting that the states after 168 h and 96 h of soaking did not differ much, indicating that the macroscopic state changes in the coating tended to be gradual, and the corrosion phenomenon did not further intensify. This phenomenon might be related to the accumulation of corrosion products, which, to some extent, acted as a barrier and slowed down the further development of corrosion [25].

**Figure 2:**
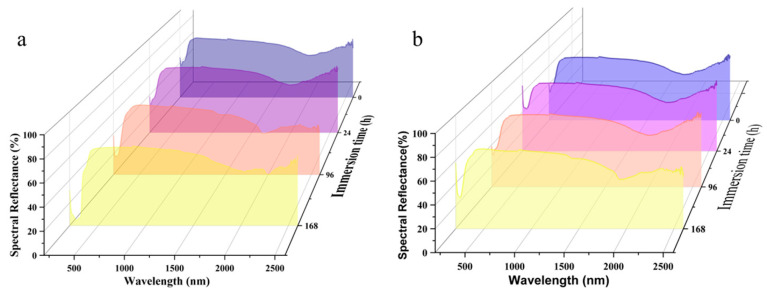
Spectral of solar absorption rate of the coating after different soaking times: (**a**) sol–gel/MAO coating; (**b**) MAO coating.

As corrosive media continue to erode the coating, the surface structure of the coating undergoes significant changes, which in turn leads to the degradation of its thermal control performance. As illustrated in Figure 2, compared with the unmodified micro-arc oxidation (MAO) coating without sol–gel sealing treatment, the sol–gel modified MAO composite coating exhibits a slower degradation rate of thermal control performance and higher structural stability under the same corrosion conditions. Specifically, the solar absorptance of the MAO coating increases from 0.32 to 0.45, while that of the sol–gel/MAO composite coating only increases from 0.32 to 0.37. This finding directly verifies that the sol–gel sealing layer can effectively inhibit the infiltration and erosion of corrosive media into the MAO coating, retard the structural damage of the coating, and thus exert a significant protective effect on the thermal control performance of the magnesium alloy MAO coating. In this paper, characterization methods such as EIS, XRD, EDS, SEM, and SEM-mapping were employed to systematically analyze the effects of sol–gel sealing treatment on the thermal control properties and corrosion resistance of micro-arc oxidation (MAO) coatings.

**Figure 3:**
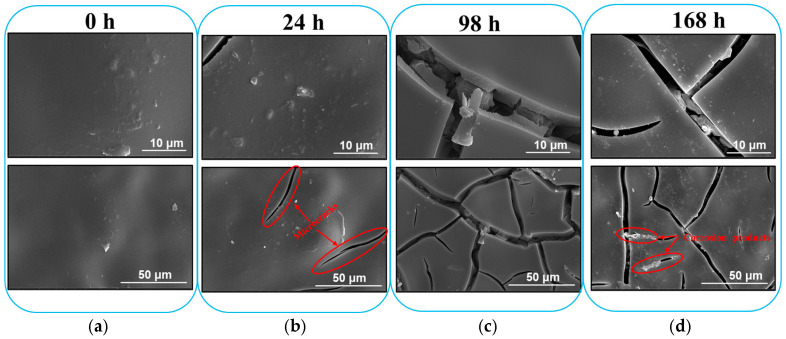
SEM morphologies of coating with different immersion times in an accelerated simulated marine environment. (**a**) 0 h, (**b**) 24 h, (**c**) 96 h, and (**d**) 168 h.

The SEM was used to systematically characterize the coating samples with different soaking times (0–168 h), as shown in Figure 3. This revealed the dynamic change process of the sol–gel coating under the corrosive environment. The SEM images of the initial state of the coating showed that the sol–gel coating was dense and uniform, completely covering the micro-arc oxidation (MAO) layer, indicating that the coating had good integrity and protective performance at the initial stage of preparation. This dense structure provided an effective physical barrier for the substrate, preventing the penetration of corrosive media [13,26,27]. After 24 h of soaking, the sol–gel coating began to show slight signs of corrosion, but the overall structure still maintained its integrity. The SEM images showed that there were minor local erosions on the coating surface, which might be caused by the gradual penetration of the corrosive media through the micro-pores or defects in the coating. Nevertheless, the MAO layer was still not exposed, indicating that the sol–gel coating still had certain protective ability in the short term.

After 96 h of immersion, the corrosion phenomenon significantly intensified. The SEM images showed that obvious cracks appeared on the surface of the sol–gel coating, and in some areas, the underlying MAO film layer was exposed. According to reports, this structural damage might be caused by the continuous diffusion of the corrosive medium within the coating, leading to the gradual failure of the interface between the coating and the substrate [1,28,29]. The formation of cracks further accelerated the penetration of the corrosive medium, thereby intensifying the degradation process of the coating. However, after 168 h of immersion, although the cracks of the sol–gel coating were more obvious, the MAO film layer remained intact, and no further severe damage was observed. This indicates that although the sol–gel coating underwent significant degradation after long-term immersion, the MAO film layer, as a second protective barrier, still provided certain protection for the substrate. This discovery highlights the advantages of the dual-layer coating system in corrosive environments; that is, even if the outer layer coating fails, the inner layer coating can still continue to perform its protective function [30,31].

**Figure 4:**
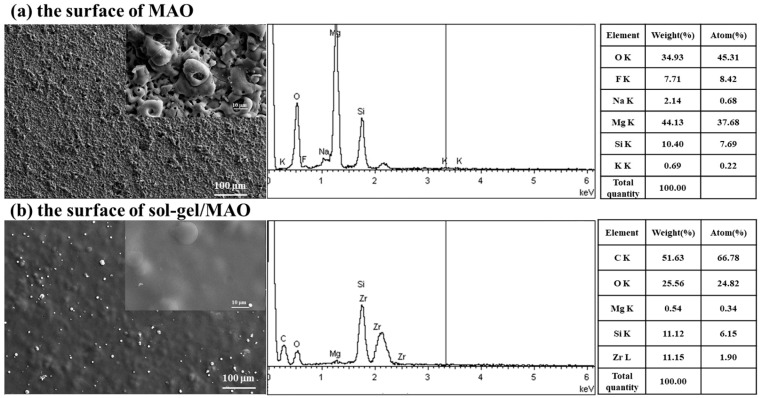
SEM images and EDS spectrum of coatings: (**a**) the surface of MAO;(**b**) the surface of sol–gel/MAO.

To clarify the differences between the MAO coating and the sol–gel/MAO composite coating, SEM and EDS were used to investigate the surface morphology and elemental composition of the coatings. Figure 4 shows the surface structure and elemental content of the MAO coating and the sol–gel/MAO composite coating. Figure 4a reveals that the porosity of the MAO coating surface is relatively high, and molten oxide particles with different sizes are randomly distributed on the coating surface. As shown in previous studies, the pores are formed by the molten oxide and bubbles thrown out in the micro-arc discharge channel, while microcracks are caused by the rapid solidification of the molten oxide in the relatively cooled electrolyte under the effect of thermal stress [32,33]. These pores are the transmission channels for reactants and products during the micro-arc oxidation process, but the high-porosity layer allows corrosive ions to penetrate the magnesium alloy substrate and continue the corrosion process [26,34,35].

Figure 4b shows the surface morphology of the sol–gel/MAO composite coating. It can be clearly observed that a continuous, uniform, and high-flatness sol–gel film is formed on the surface of the micro-pores and micro-cracks of the MAO coating, which initially indicates that the sol–gel layer can effectively cover and seal the surface defects of the MAO coating. The EDS spectra of the MAO coating and sol–gel/MAO composite coating on the magnesium alloy substrate are shown in Figure 4a and Figure 4b, respectively. The results in Figure 4a indicate that the MAO coating mainly contains elements such as O (0.5 keV), F (0.7 keV), Mg (1.3 keV), and Si (1.8 keV), corresponding to its own phase composition. Figure 4b is the EDS spectrum of the sol–gel/MAO composite coating, in which significant characteristic peaks of the sol phase (Si Kα and Zr Kα) appear. The sol–gel layer has high contents of Si and Zr, which exhibit high-intensity characteristic peaks at 1.8 keV and 2.1 keV, respectively, confirming that the sol–gel layer is successfully deposited on the surface of the MAO coating. In addition, a large amount of C element is detected in the EDS spectrum, which is mainly derived from the organic binder in the gel system [1,36]. Notably, no characteristic peaks of F and Mg are observed in the EDS spectrum of the composite coating, which further confirms that the sol–gel coating is dense and uniform, capable of completely covering the MAO film and effectively sealing most of the pores and micro-cracks in the micro-arc oxidation layer.

**Figure 5:**
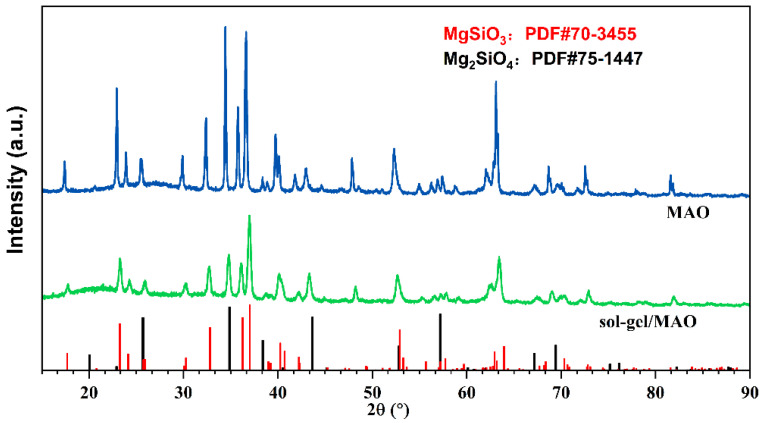
XRD spectra of MAO and sol–gel/MAO.

To investigate the material composition of the MAO coating and sol–gel/MAO composite coating, XRD tests were performed on the samples before and after sol–gel sealing, and the XRD patterns are shown in Figure 5. The XRD patterns show that the MAO coating is mainly composed of Mg_2_SiO_4_ and MgSiO_3_. Compared with the MAO coating, the sol–gel/MAO composite coating does not show obvious new diffraction peaks, but only a slight decrease in the intensity of diffraction peaks after sealing. The reason for this phenomenon is that the sol–gel sealing layer is thin and mainly exists in an amorphous form, without forming obvious crystalline phases detectable by XRD. Its weak absorption effect on X-rays leads to a slight decrease in the diffraction signal intensity of the underlying MAO coating and magnesium alloy substrate. The XRD test results are highly consistent with the EDS analysis conclusions, further confirming that the sol–gel layer has been uniformly covered on the surface of the MAO coating and achieved an effective sealing effect.

**Figure 6:**
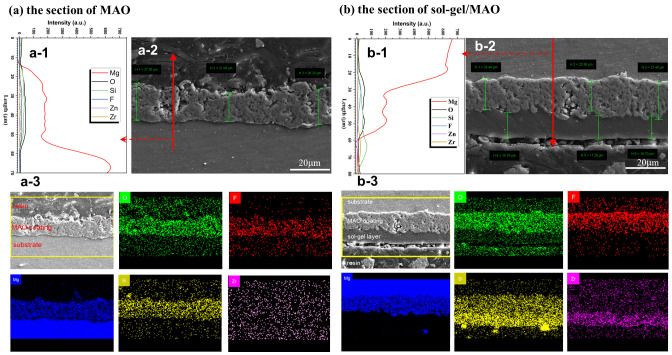
(**a-1**) EDS line scanning of the MAO coating cross-section; (**a-2**) SEM images of the MAO coating cross-section; (**a-3**) mapping of the MAO coating cross-section; (**b-1**) EDS line scanning of the sol-gel/MAO coating cross-section; (**b-2**) SEM images of the sol-gel/MAO coating cross-section; (**b-3**) mapping of the sol-gel/MAO coating cross-section.

Figure 6 shows the cross-sectional morphologies and line-scan energy-dispersive X-ray spectroscopy (EDS) characterizations of the MAO coating and the sol–gel/MAO composite coating. The longitudinal structural features and elemental distributions of the two coatings can be clearly observed. Figure 6(a-1,b-1) present the line-scan EDS profiles of the cross-sections of the MAO coating and the sol–gel/MAO composite coating, respectively, reflecting the elemental composition from the substrate to the coating surface along the longitudinal direction. In Figure 6(a-1), the elemental distribution across the MAO coating can be observed: the Mg content gradually decreases from the substrate toward the surface, while the O content first increases and then decreases, indicating that the inner dense layer is mainly composed of magnesium oxide. In the region near the outer porous layer, the contents of silicon (Si) and fluorine (F) increase gradually, suggesting that silicate and fluoride ions in the electrolyte decompose and participate in coating formation under the high-temperature and high-pressure conditions of micro-arc discharge. In Figure 6(b-1), distinct characteristic peaks of Si and Zr can be observed in the sol–gel layer. Combined with the cross-sectional elemental mapping of the sol–gel/MAO coating in Figure 6(b-3), it can be seen that the Si-Zr precursor forms a hybrid film with uniform elemental distribution after sol–gel curing and heat treatment. Figure 6(a-3) displays the elemental mapping of the MAO coating cross-section, showing that the Si content is significantly higher near the outer porous layer than near the substrate, consistent with the MAO line-scan results.

Figure 6(a-2) reveals that the cross-sectional thickness of the MAO coating is approximately 25.1 μm, and the coating is clearly composed of an outer porous layer and an inner dense layer. Surface pores are typical characteristics resulting from micro-arc discharge during the MAO process. Although these pores help improve the mechanical bonding strength between the MAO coating and the substrate, they also act as pathways for corrosive medium penetration, thereby impairing the long-term protective performance of the coating. In contrast, cross-sectional analysis of the sol–gel/MAO composite coating in Figure 6(b-2) indicates that the composite coating consists of a sol–gel sealing layer, an outer porous layer, and an inner dense layer. The thickness of the sol–gel film is approximately 17.1 μm, which effectively seals the pores generated during MAO treatment and significantly improves coating density. The sol–gel-treated coating surface is extremely smooth, which helps reduce surface defects and thus lowers the probability of direct contact between corrosive media and the substrate. However, the sol–gel film is relatively thin in some local regions, which may become weak sites for corrosive medium infiltration. This reasonably explains the localized corrosion observed in the subsequent immersion tests.

During the micro-arc oxidation process, silicate ions (SiO_3_^2−^) and fluoride ions (F^−^) in the electrolyte adsorb onto the coating surface via electrochemical reactions and diffuse inward gradually as the coating grows. In the outer porous layer, which is in direct contact with the electrolyte, the concentrations of Si and O are higher. Combined with previous studies and the present XRD, EDS, and SEM mapping results, the MAO coating is inferred to be mainly composed of MgO, MgSiO_3_, Mg_2_SiO_4_, and MgF_2_ phases [21]. The formation of MgSiO_3_ and Mg_2_SiO_4_ requires a large amount of Si, and such phases tend to preferentially form in the outer region of the coating. At the MAO coating interface, EDS line scanning shows that the Mg content in the outer layer gradually decreases, whereas the Si and O contents show no obvious decline. Combined with the XRD results, it can be deduced that in the early stage of coating formation, anodic oxidation mainly produces MgO. After MgO grows to a certain thickness, the micro-arc oxidation reaction is initiated, and silicate and fluoride ions in the solution participate in the reaction, mainly forming Mg_2_SiO_4_ and MgF_2_. However, no obvious diffraction peak of MgF_2_ appears in the XRD pattern owing to its low crystallinity.

**Figure 7:**
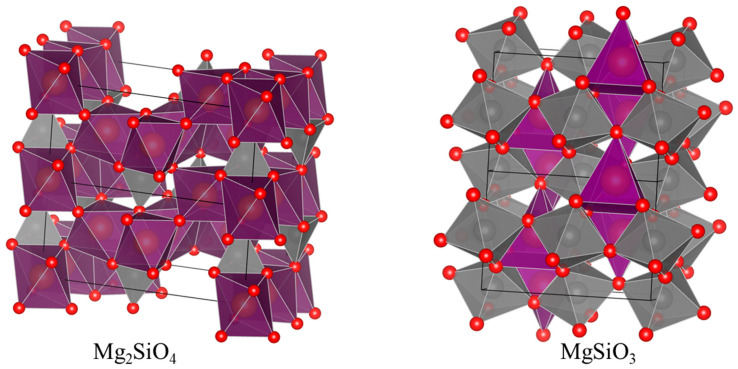
The magnesium olivine crystal phase of Mg_2_SiO_4_ and the perovskite-like crystal phase of MgSiO_3_. (Note: Detailed information about the crystal-related materials is obtained from the “Materials Project” database and was modeled using the VESTA 3.90.6a software).

Based on the results of cross-sectional linear EDS scanning and surface XRD characterization, it can be reasonably speculated that the available surface Mg^2+^ concentration gradually declines with the continuous progression of the coating growth reaction, which drives the bulk Mg/Si stoichiometric ratio of the ceramic coating to gradually evolve from 2:1 toward 1:1. Correspondingly, the film-forming phase tends to transform from Mg-rich island-shaped silicate (Mg_2_SiO_4_, forsterite phase) to Si-dominated chain-structured silicate (MgSiO_3_, perovskite-like phase), and the two distinct crystal structures are displayed in Figure 7. This hypothesized phase transition is expected to enhance the polymerization degree of the siloxane framework. Specifically, the isolated (SiO_4_)^4−^ tetrahedra in the original forsterite phase may convert into (SiO_3_)^2−^ single-chain configurations of the perovskite-like phase, which increases the proportion of Si-O-Si bridging oxygen and distorts the local coordination environment of Mg^2+^ ions [37,38].

According to the band theory, the rearrangement of bridging oxygen may lower the energy of the O 2p orbitals and change the crystal field environment, thereby causing the maximum value of the valence band to shift downward [39,40]. Combined with the optical absorption edge formula, this expansion of the band gap may cause the intrinsic absorption edge to shift toward the blue. For an ideal single-phase crystal, this structural evolution is likely to weaken the intrinsic light absorption capability within the visible light–near-infrared region (400–2500 nanometers), thereby leading to a decrease in the solar absorption rate (α_S_).

Meanwhile, the variation in infrared emissivity (ε) is primarily governed by lattice vibration behaviors. It is inferred that the chain structure of the evolved perovskite-like phase can convert the discrete, sharp phonon modes of the initial forsterite phase into broadband continuous vibrations with effective interchain coupling. This evolution significantly broadens the phonon density of states, especially within the critical atmospheric window band of 8–14 μm, which ultimately promotes an increase in infrared emissivity. In summary, the speculated phase transition from forsterite to perovskite-like structure modulates the electronic structure of the coating to reduce solar absorptance, while reconstructing lattice vibrational modes to elevate infrared emissivity [39].

**Figure 8:**
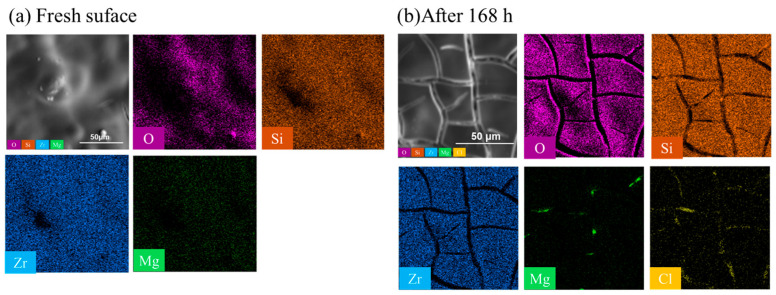
SEM-mapping: (**a**) fresh surface of the sol–gel/MAO composite coating; (**b**) the sol–gel/MAO composite coating after being immersed in a 3.5% NaCl solution simulating the coastal environment for 168 h.

Figure 8 shows the surface elemental distributions of the sol–gel/MAO composite coating before and after 168 h of immersion. As shown in Figure 8a, SEM-mapping of the as-prepared sol–gel/MAO composite coating reveals that zirconium, silicon, and oxygen are uniformly distributed on the fresh surface, whereas the signal of magnesium is relatively weak. This observation further confirms that the sol–gel layer forms an effective protective barrier on the MAO coating surface, which is consistent with the previous EDS analysis results. As displayed in Figure 8b, obvious cracks appear on the coating surface after 168 h of accelerated corrosion in a simulated coastal environment. From the perspective of formation mechanism, the sol–gel coating is fabricated via a series of chemical reactions (hydrolysis, condensation, drying, and low-temperature curing) involving the combination of Zr and Si precursors with organic binders. During the preparation process, the sol–gel precursors undergo hydrolysis and polycondensation reactions to form a three-dimensional network structure. After drying and low-temperature curing, this network structure is further densified, forming a compact and continuous sealing layer on the surface of the MAO coating.

The formation of this dense sealing layer is the primary mechanism responsible for the improved corrosion resistance of the sol–gel/MAO composite coating compared to the single MAO coating. For the single MAO coating, the inherent porous structure and micro-cracks provide direct channels for the penetration of corrosive media, leading to the gradual corrosion of the magnesium alloy substrate. In contrast, the sol–gel sealing layer not only fully covers the surface of the MAO coating but also penetrates into the pores and micro-cracks of the MAO layer, effectively blocking the diffusion paths of corrosive media (e.g., water and chloride ions) to the substrate. The Zr and Si components in the sol–gel layer form stable chemical bonds (e.g., Si-O-Si and Zr-O-Si) during curing, which improve the structural stability and compactness of the coating, further improving its barrier performance against corrosive media. The weak Mg signal on the initial coating surface directly verifies that the sol–gel layer effectively isolates the MAO coating and the magnesium alloy substrate from the external environment, laying a foundation for its excellent corrosion resistance during long-term immersion.

### 3.2. Electrochemical Behaviors

#### 3.2.1. Potentiodynamic Polarization

**Figure 9:**
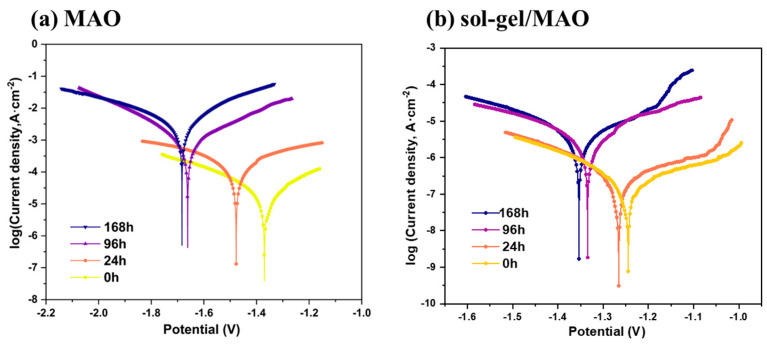
Potentiodynamic polarization curves of different immersion times in 3.5 wt.% NaCl solution: (**a**) MAO; (**b**) sol–gel/MAO.

**Figure 10:**
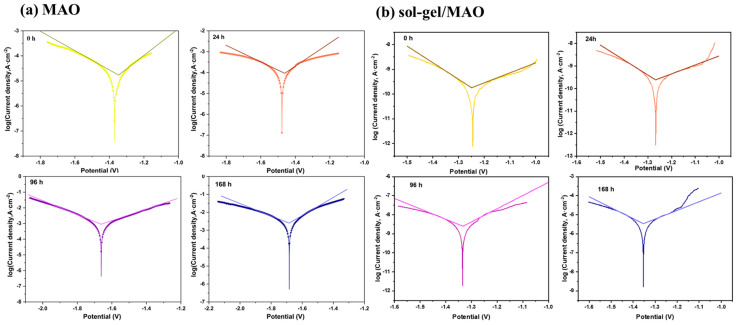
Tafel fitting curves of the electromotive force polarization curves at different soaking times: (**a**) MAO; (**b**) sol–gel/MAO.

The polarization curves of MAO and sol–gel/MAO coatings after different immersion durations are presented in Figure 9, and Figure 10 displays the Tafel regions derived from the corresponding polarization curves via Tafel extrapolation. This fitting method is adopted because it serves as a classic and well-established technique in electrochemical corrosion research for calculating critical corrosion parameters, including corrosion current density (*i*_corr_) and corrosion potential (E_corr_), and it is suitable for the quantitative analysis of corrosion behaviors of composite coatings in this work [41]. The anodic and cathodic slopes, corrosion potential, and corrosion current obtained by linear fitting of polarization zones are summarized in Table 2. Compared with unimmersed specimens, immersed sol–gel/MAO and MAO samples exhibit higher corrosion current and lower corrosion resistance, revealing a negative correlation between immersion time in 3.5 wt% NaCl solution and coating corrosion resistance. The corrosion potential follows the same variation tendency. In terms of corrosion current, the value of sol–gel/MAO coating slightly rises from 10^−6.4^ A/cm^2^ to 10^−6.2^ A/cm^2^ after 24 h immersion, indicating mild aggravation of corrosion reaction, which coincides with the morphological evolution observed by electron microscopy. By contrast, the corrosion current of MAO coating increases from 10^−4.8^ A/cm^2^ to 10^−4.2^ A/cm^2^. This result demonstrates that the sol–gel layer delivers superior anti-corrosion performance at the early immersion stage. After 96 h immersion, the corrosion current of sol–gel/MAO coating increases remarkably from 10^−6.4^ A/cm^2^ to 10^−5.2^ A/cm^2^, reflecting a substantial deterioration in corrosion resistance, which is closely associated with the fracture of the sol–gel film observed microscopically. Meanwhile, the current of MAO coating rises from 10^−4.8^ A/cm^2^ to 10^−3.1^ A/cm^2^, suggesting severe corrosion damage to the coating.

However, when comparing the subsequent immersion of 168 h with 96 h, the changes in the self-corrosion current and potential are not significant, which also proves that the MAO coating is the main factor in blocking the corrosion medium from penetrating into the substrate in the later stage of immersion. Due to the corrosion products covering the reaction area that prevents corrosion (anodic area), the anodic resistance (b_a_) of the sample increases after 96 h. However, the cathodic area remains exposed to the solution, and as the time increases from 0 h to 24 h, the continuous reaction causes the cathodic reaction resistance (b_c_) to decrease from 6.7 V/decade to 5.47 V/decade [42].

#### 3.2.2. Electrochemical Impedance Spectroscopy

**Figure 11:**
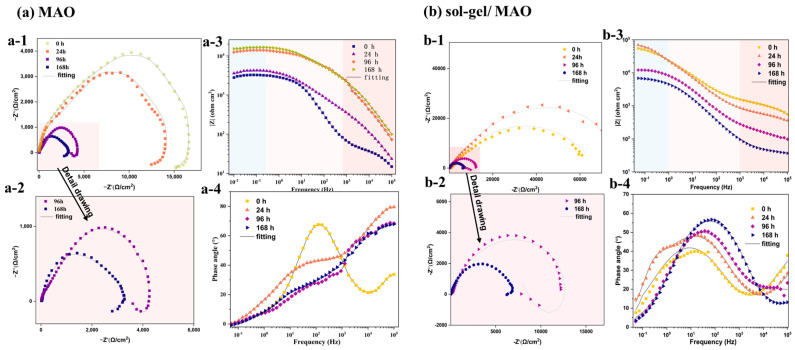
Experimental and fitting results of EIS of (**a**) sol–gel/MAO composite coating and (**b**) sol–gel/MAO coating on MB15 magnesium alloy after different immersion times in 3.5 wt% NaCl solution: (1) Nyquist plot, (2) Nyquist detail plot, and (3) and (4) Bode plots.

The impedance spectra shown in Figure 11 include experimental data (indicated by symbols) and theoretical fitting curves (solid lines). By comparing the EIS spectra before and after sol–gel sealing, it can be seen that the impedance modulus of the pure MAO coating in Figure 11(a-1,a-2) is much lower than that of the sol–gel/MAO composite coating in Figure 11(b-1,b-2), demonstrating that the sol–gel sealing treatment effectively improves the anti-corrosion performance of the coating. In the Nyquist plot of Figure 11(b-1), all samples exhibit a single capacitive arc, whose radius continuously decreases with the increase in immersion time, which reflects a significant decline in corrosion resistance. The Bode impedance plot in Figure 11(b-2) further reveals that the low-frequency impedance modulus (|Z|, within the range of 0.01–1 Hz) gradually decreases as the exposure time extends. Within the first 48 h, the coating maintains a high modulus value, indicating that the coating still has effective barrier performance. However, after this period, |Z| drops by an order of magnitude, also indicating a severe degradation of the coating’s corrosion resistance.

**Figure 12:**
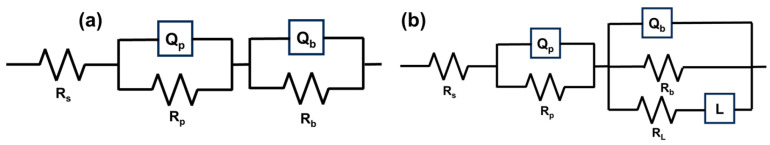
Equivalent circuits of the EIS plots for the MAO coating with a long-time EIS test. (**a**) Immersion time 0–24 h; (**b**) immersion time after 24 h.

According to the analysis in Figure 11(b-4), two time constants can be observed in the Bode phase diagram of the samples before 48 h of immersion. Therefore, the equivalent circuit shown in Figure 12a was used for fitting. In the Bode phase diagrams of other sealed samples, only one times constant was observed, so the equivalent circuit shown in Figure 12b was used for fitting. Here, the electrical components consist of resistive elements (R_p_ represents the polymer layer, R_b_ represents the MAO layer, and R_L_ and L represent the resistance and inductance associated with pitting corrosion) and constant phase elements (Q_p_ and n_p_ represent the polymer layer, and Q_b_ and n_b_ represent the MAO layer. (n_P_ and n_B_ are two dispersion factor parameters belonging to constant phase element *Q*. The dispersion coefficient n ranges between 0 and 1, which describes the deviation degree from ideal capacitor behavior. When (n = 1), CPE (Q) behaves identically to ideal capacitor C; when (n = 0), Q is equivalent to pure resistance.) [43]. From the results of Q_p_ and R_p_, it can be seen that as the immersion time in the sodium chloride solution increases, the admittance of the polymer CPE increases from 5.36 × 10^−7^ Ω^−1^·cm^−2^·S^−n^ to 2.92 × 10^−5^ Ω^−1^·cm^−2^·S^−n^, and the reaction resistance of the polymer decreases from 8.66 × 10^4^ to 6.78 × 10^3^ Ω·cm^−2^. This indicates a sharp decline in the corrosion resistance of the polymer film of the sample.

It is worth noting that a capacitive loop appears in the Nyquist plot of the sample after 168 h of immersion in Figure 11a, indicating that as the immersion time increases, the corrosive medium penetrates the composite coating and invades the metal substrate, initiating local pitting corrosion. This change in impedance response highlights the staged degradation process of the composite coating under continuous corrosion. From the fitting results in Table 3, the appearance of an inductive loop after 168 h also indicates the occurrence of pitting. Combined with the SEM morphology analysis in Figure 2, corrosion of the sample in the NaCl solution begins after 96 h of immersion, and the initial sol–gel polymer layer still maintains a flat and dense morphology. From the cross-sectional SEM image in Figure 6, it can be seen that after the sol–gel polymerization reaction occurs on the MAO layer, local weak areas are formed, with a thickness less than the average. As the corrosive medium continues to invade, it begins to break through the weak areas of the polymer, and microcracks start to appear in the polymer layer of the composite coating, exposing the internal MAO layer. The above corrosion process is in good agreement with the EIS fitting results.

## 4. Conclusions

This study tested the failure process of sol–gel/MAO composite coatings on MB15 magnesium alloy during long-term soaking. We used multiple test methods to analyze the samples. We found that the corrosion resistance of the coating changes with its structure. The main results are as follows:

(1) We prepared the sol–gel/MAO composite coating on MB15 magnesium alloy. This coating keeps good thermal control performance and protects the sample well in high-salt and high-humidity environments. The composite structure slows down the corrosion product generation process and delivers enhanced anti-corrosion capability.
(2) The sol–gel layer seals the micro-pores and cracks of the MAO coating effectively. It blocks the penetration of corrosive media, so the composite coating has obviously enhanced anti-corrosion ability compared to the single MAO coating.
(3) The double-layer structure of sol–gel and MAO shows an observable synergistic protective tendency. It reduces the degradation of thermal control performance caused by corrosion, and it has good application potential for aerospace magnesium alloy parts.

## Figures and Tables

**Table 1:** The element content of MB15 alloy (wt%).

Element	Zn	Zr	Al	Si	Mg
Content	5.46	0.64	<0.05	<0.05	Balance

**Table 2:** The fitting calculation results for the linear polarization zone of polarization curves.

Samples	Time (h)	b_a_ (V/Decade)	b_c_ (V/Decade)	*i*_corr_ (A/cm^2^)	E_corr_ (V)
MAO	0	5.35 ± 0.11	−3.85 ± 0.22	10^−4.8^ ± 10^−0.12^	−1.35 ± 0.005
	24	5.61 ± 0.23	−3.97 ± 0.11	10^−4.1^ ± 10^−0.20^	−1.45 ± 0.006
	96	3.72 ± 0.26	−4.45 ± 0.34	10^−3.1^ ± 10^−0.09^	−1.66 ± 0.007
	168	5.04 ± 0.08	−3.47 ± 0.35	10^−2.8^ ± 10^−0.40^	−1.68 ± 0.031
Sol–gel/MAO	0	4.02 ± 0.22	−6.70 ± 0.28	10^−6.4^ ± 10^−0.08^	−1.25 ± 0.024
	24	3.96 ± 0.09	−6.63 ± 0.14	10^−6.2^ ± 10^−0.14^	−1.27 ± 0.004
	96	6.91 ± 0.24	−5.47 ± 0.55	10^−5.2^ ± 10^−0.19^	−1.33 ± 0.007
	168	4.56 ± 0.15	−5.75 ± 0.24	10^−5.1^ ± 10^−0.31^	−1.35 ± 0.009

**Table 3:** Fitted data by Zsimpwin of EIS results.

Immersion Time (h)	R_s_ (Ω/cm^2^)	Q_p_ (Ω^−1^s^n^ cm^−2^)	n_p_	R_p_ (Ω/cm^2^)	Q_b_ (Ω^−1^s^n^ cm^−2^)	n_b_	R_b_ (Ω/cm^2^)	R_L_ (Ω/cm^2^)	L (Ω^−1^s^n^ cm^−2^)
0	13.8	5.36 × 10^−7^	0.58	8.66 × 10^4^	8.40 × 10^−7^	0.60	1274	-	-
24	5.6	1.09 × 10^−5^	0.62	6.25 × 10^4^	1.03 × 10^−5^	0.57	593.3	-	-
96	2.4	1.59 × 10^−5^	0.75	1.02 × 10^4^	6.48 × 10^−4^	0.23	27	1.18 × 10^9^	1.11 × 10^4^
168	10.1	2.92 × 10^−5^	0.71	6785	8.44 × 10^−7^	0.67	28	41	342

## Data Availability

The original contributions presented in this study are included in the article. Further inquiries can be directed to the corresponding authors.
